# Dynamics of chromatin accessibility and genome wide control of desiccation tolerance in the resurrection plant *Haberlea rhodopensis*

**DOI:** 10.1186/s12870-023-04673-2

**Published:** 2023-12-19

**Authors:** Petko Mladenov, Xiaohua Wang, Zhaolin Yang, Dimitar Djilianov, Xin Deng

**Affiliations:** 1 State Key Laboratory of Plant Diversity and Specialty Crops, Institute of Botany, Chinese Academy of Sciences, Beijing, 100093 China; 2 Agricultural Academy, 8 Dragan Tzankov Blvd, Sofia, 1164 Bulgaria; 3 China National Botanical Garden, Beijing, 100093 China; 4 University of Chinese Academy of Sciences, Beijing, 100049 China

**Keywords:** Resurrection plants, Desiccation tolerance, ATAC-seq, Epigenetic regulation, Transcription factors, Gene expression

## Abstract

**Background:**

Drought is one of the main consequences of global climate change and this problem is expected to intensify in the future. Resurrection plants evolved the ability to withstand the negative impact of long periods of almost complete desiccation and to recover at rewatering. In this respect, many physiological, transcriptomic, proteomic and genomic investigations have been performed in recent years, however, few epigenetic control studies have been performed on these valuable desiccation-tolerant plants so far.

**Results:**

In the present study, for the first time for resurrection plants we provide evidences about the differential chromatin accessibility of *Haberlea rhodopensis* during desiccation stress by ATAC-seq (Assay for Transposase Accessible Chromatin with high-throughput sequencing). Based on gene similarity between species, we used the available genome of the closely related resurrection plant *Dorcoceras hygrometricum* to identify approximately nine hundred transposase hypersensitive sites (THSs) in *H. rhodopensis*. The majority of them corresponds to proximal and distal regulatory elements of different genes involved in photosynthesis, carbon metabolism, synthesis of secondary metabolites, cell signalling and transcriptional regulation, cell growth, cell wall, stomata conditioning, chaperons, oxidative stress, autophagy and others. Various types of binding motifs recognized by several families of transcription factors have been enriched from the THSs found in different stages of drought. Further, we used the previously published RNA-seq data from *H. rhodopensis* to evaluate the expression of transcription factors putatively interacting with the enriched motifs, and the potential correlation between the identified THS and the expression of their corresponding genes.

**Conclusions:**

These results provide a blueprint for investigating the epigenetic regulation of desiccation tolerance in resurrection plant *H. rhodopensis* and comparative genomics between resurrection and non-resurrection species with available genome information.

**Supplementary Information:**

The online version contains supplementary material available at 10.1186/s12870-023-04673-2.

## Introduction

Global climate changes affect agricultural production. Recent expansion of semiarid regions and proposed models suggest that drought conditions may become longer and more severe [1] and thereby demanding research in the field of drought response and crop biotechnology. In evolution, different mechanisms have been developed by higher plants to reduce the negative impact of drought. Some species known as ‘resurrection plants’ show tolerance to desiccation—an extreme state of drought stress where cellular water is almost entirely depleted (complete dehydration, > 90% water loss). The vegetative tissues of resurrection plants can survive desiccation and subsequently recover rapidly upon rehydration [2]. In this respect, they provide an excellent model toward understanding drought tolerance and utilizing their evolutionary benefits [2, 3].

Resurrection plants represent a very small part of the common flora, spread in different geographical regions and belonging to different families of angiosperms [4, 5]. The Gesneriaceae family is the richest source of resurrection plants among dicot families. It comprises over 3200 species that are distributed almost all around the world and systematically classified into several subfamilies and tribes [6]. Desiccation tolerance has been reported only for 16 species, all of which belong to the Trichosporeae tribe in Didymocarpoideae subfamily, including *Boea hygrometrica* (now also known as *Dorcoceras hygrometricum*) widely distributed in China and *H. rhodopensis* located in the Balkan Peninsula [7, 8]. So far the only genome sequenced resurrection species of Gesnereaceae is *B. hygrometrica* (Hereafter, it will be referred to as *D. hygrometricum*) [9]. The *D. hygrometricum* genome sequence information has provided a suitable reference for transcriptomics and proteomics studies in Gesnereaceae resurrection plants.

*H. rhodopensis* has been studied extensively through various physiological, metabolomic, transcriptomic and proteomic studies to decipher its desiccation tolerance [10–15]. Different mechanisms of adaptations related with photosynthesis have been discovered including changes in the structure and protein composition of the photosynthetic machinery. These changes result in a reduction of light harvesting during desiccation, leading to a shift from linear electron flow (LEF) to cyclic electron flow (CEF) preventing the over reduction of electron transport chains due to the decreased carbon assimilation during desiccation. Consequently, CEF becomes the primary source for generating ATP and maintaining the ATP/NAPDH ratio in *H. rhodopensis* dehydrated in the light [11, 13]. Accumulation of various enzymatic and non-enzymatic scavengers has also been observed during dehydration, however, some evidences exist about generation of ROS during dehydration such as increasing of MDA levels and damaging and degradation of some cellular components such as chloroplasts, as observed in other resurrection plants [15–17]. These processes have also been accompanied by increased expression of various transcripts and proteins related to ER stress and autophagy and a decreased expression of proteins involved in programmed cell death [14, 15]. The protection of cellular and molecular structures during osmotic stress is ensured mainly by the accumulation of osmolytes such as sucrose, raffinose and ɑ-tocopherol, and the expression of intrinsically disordered chaperon proteins such as heat shock proteins (HSPs), late embryogenesis abundant proteins (LEAs, including dehydrins) and Thaumatin-like proteins (TLPs) in different parts of cells, as well as proteins protecting photosynthetic apparatus such as early light inducible proteins (ELIPs), which are similar to those observed in other resurrection plants such as *D. hygrometricum* and *Craterostigma plantagineum* [2, 12–16, 18, 19]. De novo synthesis of proteins and metabolic compounds could occur under limited photosynthesis and carbon assimilation conditions, potentially due to the activation of alternative carbon sources and autophagy-derived supply from autophagy [20, 21]. This hypothesis is further supported by the observed decrease in the overall pool of branched amino acids and certain glycerophospholipids during dehydration, consistent with reports in other resurrection plants [13, 22, 23]. Moreover, a reduction in saturated fatty acids and a concurrent increase in glycerophosphodiesters during desiccation have been exclusively documented in *H. rhodopensis* [12, 13]*.* Recently, a multiomics-based model has been proposed to explain the assimilation of these compounds, taking into consideration the fluctuations of glycolytic intermediates, organic acids, and key enzymes involved in these metabolic pathways [15].

The orchestra of desiccation tolerance processes depends on the stress perception and signalling, leading to programmed activation and repression of genes in the plant genomes. The identification and characterization of highly accessible chromatin regions in plant genomes helps in understanding transcriptional regulation and location of genomic regulatory elements [24–30]. These *cis*-regulatory elements are found in open chromatin regions in nucleosomes which are highly sensitive to endonucleases or transposases. The ATAC-seq approach (Assay for Transposase Accessible Chromatin with high-throughput sequencing) utilizes a hyperactive Tn5 transposase to cause DNA cleavage and simultaneous insertion of sequencing adapters into open chromatin regions. Specific *cis*-elements in the core promoters engage RNA polymerase II and general transcription factors (TFs), while distal enhancer elements recruit both positive and negative TFs, together to orchestrate multiple signalling inputs for spatial and temporal control of the transcription of downstream coding genes. Numerous TFs participating in dehydration tolerance in various resurrection plants, including WRKYs, homeodomain leucine zipper factors, bZIPs and heat shock factors [31–33] [. Conversely, a global view of the general distribution and availability of the *cis*-regulatory elements in resurrection plant genome that are involved in desiccation tolerance is limited. Recently, desiccation-induced alterations in global methylation state of the genome of *D. hygrometricum* has been reported [34]. The findings confirm that the dynamic DNA methylation in promoter regions of certain genes indeed affects the expression of corresponding genes in different adaptation states [34]. Dynamic DNA methylation and other epigenetic modification collectively induce conformational changes in chromatin structure, thereby modulating the accessibility of specific chromatin regions to RNA polymerase II and various transcription factors that activate or repress the transcription of local genes [35]. The identification of desiccation-induced genes and proteins for DNA repair and metabolism, chromatin organization and chromosome maintenance in *H. rhodopensis*, underlines also the significance of dynamic regulation on chromatin level for desiccation tolerance [14, 15].

The aim of the present study is to evaluate the changes of highly accessible chromatin regions in *H. rhodopensis* in response to desiccation. We used ATAC-seq approach to obtain genomic information of regulatory elements and to identify their corresponding genes using the available genome of the closely related species *D. hygrometricum* [13]. Furthermore, we integrated and summarized the identified genes with their previously reported expression by RNA-seq [20]. This allowed us to unveil new insights into the dynamic regulation of open chromatin regions in relation to the gene expression and protein accumulation in response to desiccation stress in the resurrection plant *H. rhodopensis*. Moreover, comparing ATAC-seq data from syntenic regions across species has been reported as an efficient way to identify common and new gene regulatory modules [25] and thus our findings will also provide a valuable resource for more comprehensive comparative genomics studies regarding drought tolerance in plants.

## Results

### Isolation of nuclei from different states of desiccation stress of *H. rhodopensis* and preparation of ATAC-seq libraries

**Fig. 1:**
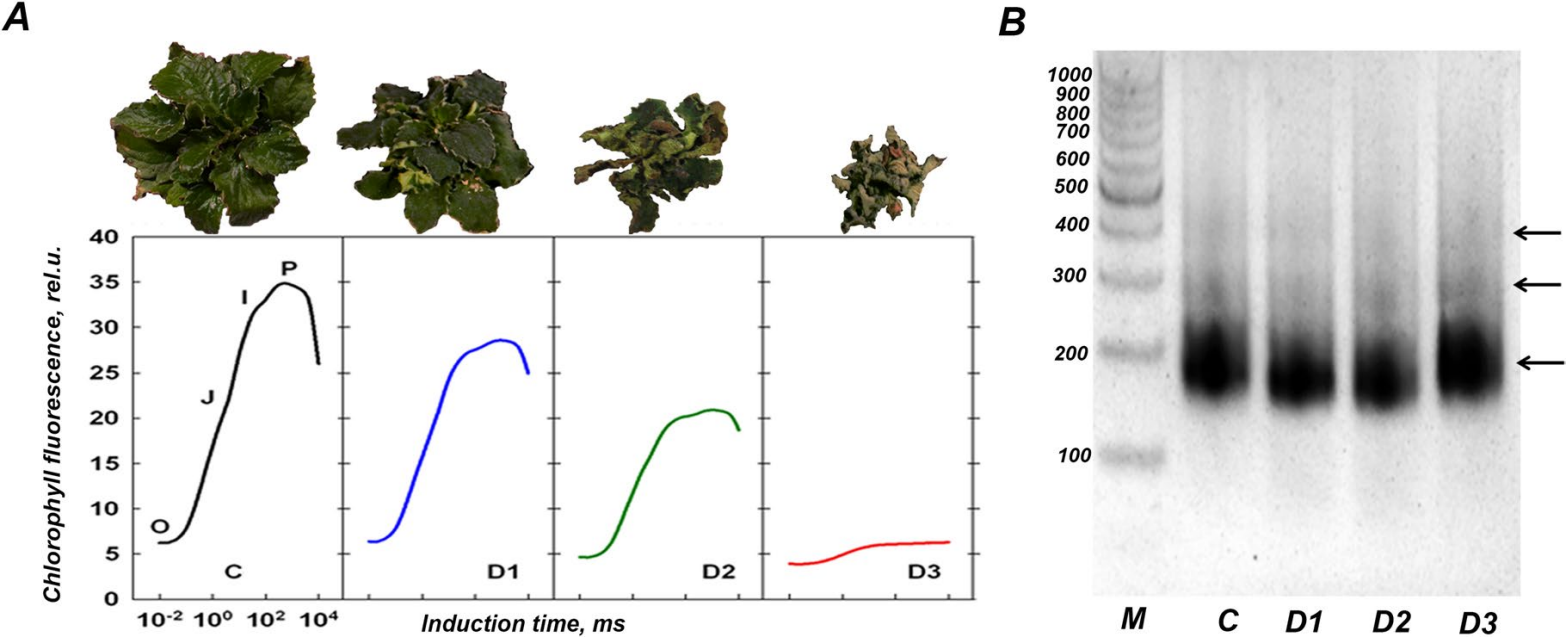
Extraction of nuclei and amplification of transposase mediated tagged nucleosome DNA from various stages of desiccation in *H. rhodopensis*. **A** Stages of desiccation in *H. rhodopensis* based on photosynthetic performance. Control (C), moderate (D1); severe (D2), and full desiccation (D3). The averaged OJIP curves of fast fluorescence yields from samples for each stage are depicted in different colours. **B** Horizontal gel electrophoresis for visualization of amplified tagged nucleosome DNA library prior to sequencing. A 2% agarose gel was used for the separation of libraries; along with a 100 bp marker (M) for reference

To identify and characterize highly accessible chromatin regions in the genome of *H. rhodopensis* during desiccation, watering was withdrawn from the plants. Leaf samples were then collected at various stress stages by evaluation and classification of photosynthetic performance, as described previously [13, 18]. Immediately after measurements of fast fluorescence on dark adapted plants, data were classified by a new trained two-layer hexagonal Self organized map (SOM) with predefined nodes corresponding to each state of stress and leaves from each stage were frozen in liquid nitrogen and stored in -80 °C for isolation of nuclei (as depicted in Fig. 1A). According to manufacturer instructions, we used 60,000 nuclei from all replicates for nucleosome tagging and amplification of ATAC libraries. The PCR program for amplification of tagged DNA was performed as described in Bajic et al. 2018 [26] without requiring additional cycles (Additional file 2). In agreement with the typical length of amplified tagmented DNA from plant genomes, we observed electrophoretic migration of the fragments in multiple bands between 180 and 500 bp (Fig. 1B).

### Sequencing of ATAC-seq libraries and data analysis

**Fig. 2:**
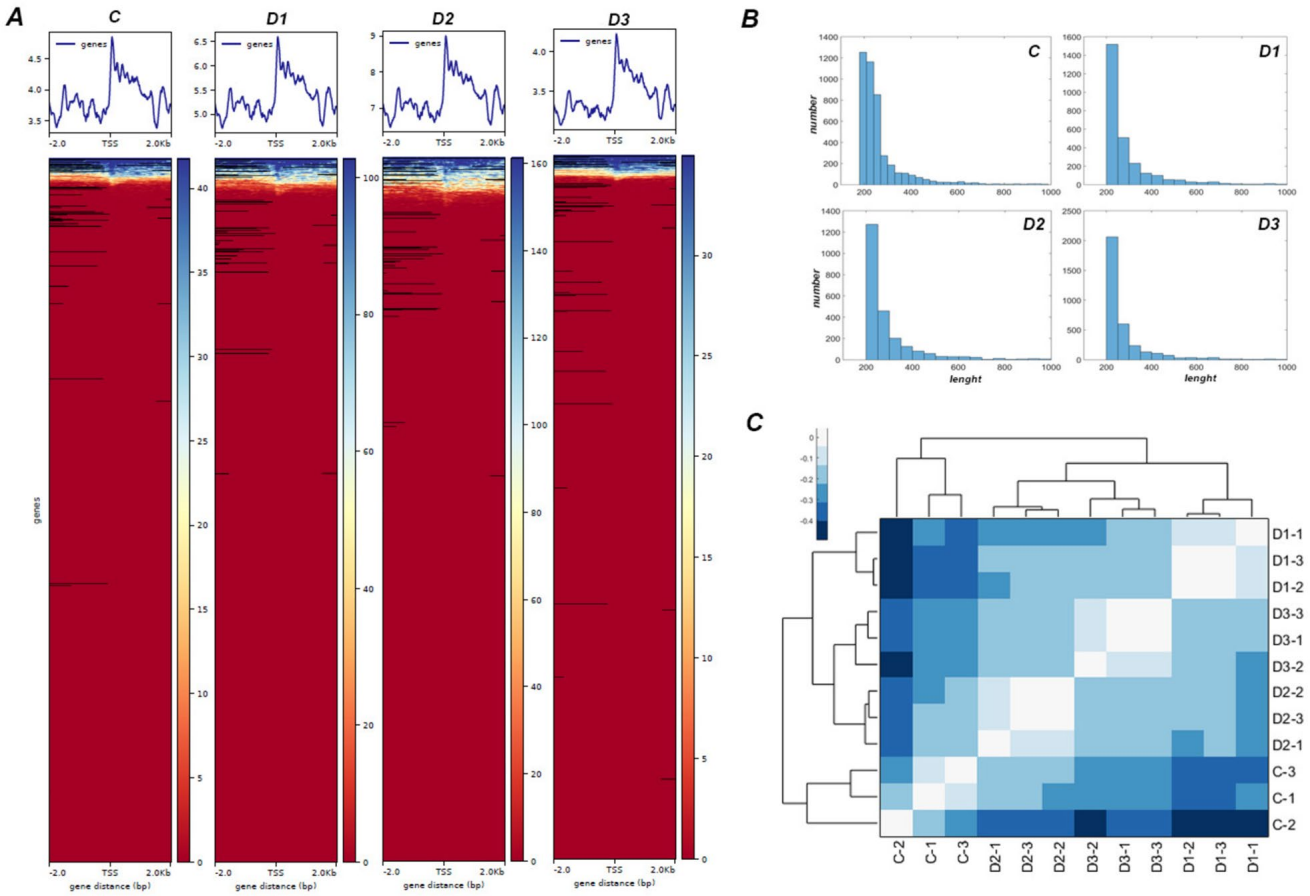
ATAC-seq datasets from various stages of desiccation of *H. rhodopensis*. **A** Representative TSS plots and heat maps for each desiccation stage, showing number of mapped sequences from the corresponding genes in *D. hygrometricum* genome. **B** Histograms of length distribution for mapped sequences in each stage of desiccation. **C** HCA-heatmap showing the log2 transformed Pearson correlation coefficients of ATAC‐seq replicates from all treatments. Replicates in unstressed state (C, control plants, fresh, watered) are differentially clustered from the samples of stressed plants, which are further grouped based on the level of desiccation stress; samples under severe desiccation (D2, partially dehydrated) and complete desiccation (D3, fully dehydrated) are distinctly separated from those under moderate stress (D1, moderate dehydrated)

Sequencing results were first evaluated by assessing the sequence length and distribution, GC content, and the distribution of base percentage and qualities along reads. An overall consistent base percentage and high base quality along reads from all replicates were observed (Additional file 3; Additional file 4). After removing Next Generation Sequencing (NGS) adapters used in amplification step and low quality reads, trimmed reads were then mapped to the published reference genome of *D. hygrometricum* [9], and duplicates generated by PCR were removed. *D. hygrometricum* is the only resurrection species closely related to *H. rhodopensis* in the same tribe (Trichosporeae) in Gesneriaceae with available genome sequencing information. The total mapped reads derived from various conditions of *H. rhodopensis* were approximately 150 million, showing similarities higher than 51% to *D. hygrometricum* counterparts. The mapped fragments corresponding to highly accessible chromatin regions from all replicates were enriched around the transcription start site (TSS) of genes, as expected; shifted between + 2 bp and − 2 bp for positive and negative strands, respectively. Most of the ATAC signals were distributed predominately between 0 – 1 kb upstream of the transcription start site (Additional file 5; Fig. 2A). The size of all mapped fragments for each treatment was represented by the distribution plot (Fig. 2B), which shows the typical ATAC-seq periodic decrease in the number of regions with the corresponding length between 200 and 800 bp. The reproducibility of experiment between biological replicates and treatments was evaluated by correlation analysis of the fold change count peak matrix of all replicates and visualised by HCA-heatmap (Fig. 2C). Higher correlation between replicates of each treatment determined their clustering altogether in clustergram, while treatments were separated according to differences between mapped nucleosome-free regions. Replicates in unstressed state (C) were differentially clustered from desiccation-stressed plants, which are grouped according to the degree of stress; samples in the severe dehydration (D2) and complete desiccation (D3) are separated from those in moderate stress (D1).

### Peak annotation and motif enrichment

**Fig. 3:**
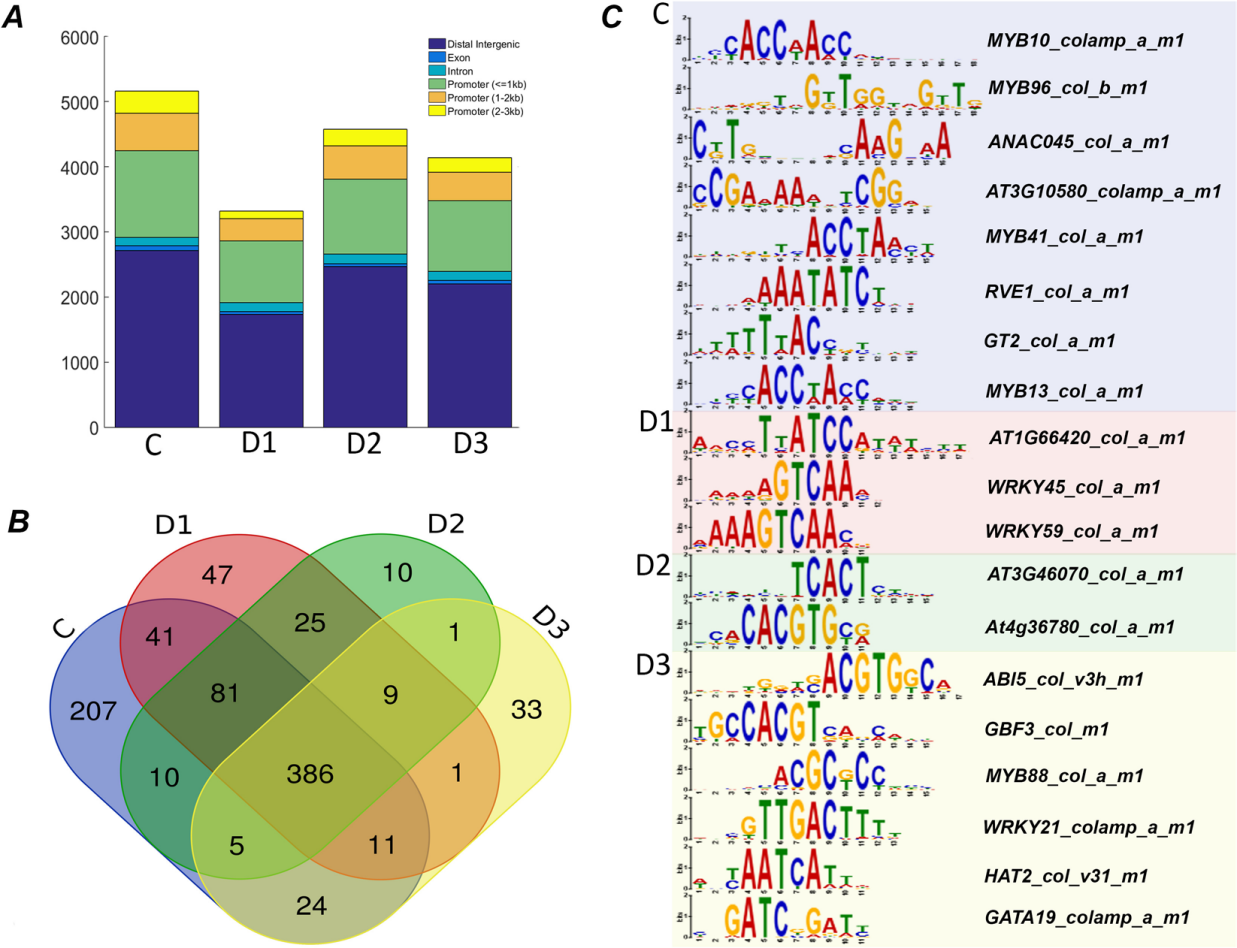
Annotation of peaks from ATAC-seq datasets and motif enrichment in promoters of THSs in different states of desiccation. **A** The number of peak counts of annotated THSs for each stress stage in *H. rhodopensis*. Colour bar represents annotation of elements according to their position of the corresponding gene. **B** Venn diagram illustrating distribution of annotated THSs among stress treatments. **C** Motif analysis in promoters of THSs found only in control, D1, D2 and D3 state. The names of the interacting transcription factors in database are given on the right panel

In our study, we annotated a total of 893 transposase hypersensitive sites (THSs) among the replicates of different desiccation states of *H. rhodopensis* (Additional file 6). Based on their proximity to the CDS region, the identified THSs were prominently found in distal intergenic regions, followed by various regions of promotor, and much less frequently in exon, intron and intragenic regions (Fig. 3 A). The overall counts of THSs differed significantly among each treatment (Fig. 3 A). The Venn diagram of the distribution of THSs in each dehydration treatment reveals that the largest number of unique elements are found in fresh samples (C, 207), followed by D1 (47), D3 (33) and D2 (10) (Fig. 3B). Total of 386 THSs were commonly found in all samples: 41 THSs were found in both C and D1; 81 were shared in C, D1 and D2; and 9 were exclusively shared among stressed plants. To better characterize the THSs unique to C, D1, D2 and D3 states, respectively, we performed sequence-based prediction for motif frequency of promotor elements (Additional file 7, Fig. 3C). This analysis resulted in the enrichment of 8 motif sequences unique for fresh plants (C), including binding sites for MYB and MYB-like transcription factors and Homeodomain-like transcription factors*.* Two specific WRKY binding sites and one DNA-binding storekeeper protein-related transcriptional regulator were enriched from D1-unique THSs. Two motifs for binding of C2H2-type zinc finger family protein were enriched from D2-unique THSs. From D3-unique THSs, binding motifs of bZIP (ABI5), homeodomain (HAT2), WRKY (WRKY21), MYB and GBF factors were enriched.

### Functional annotation

**Fig. 4:**
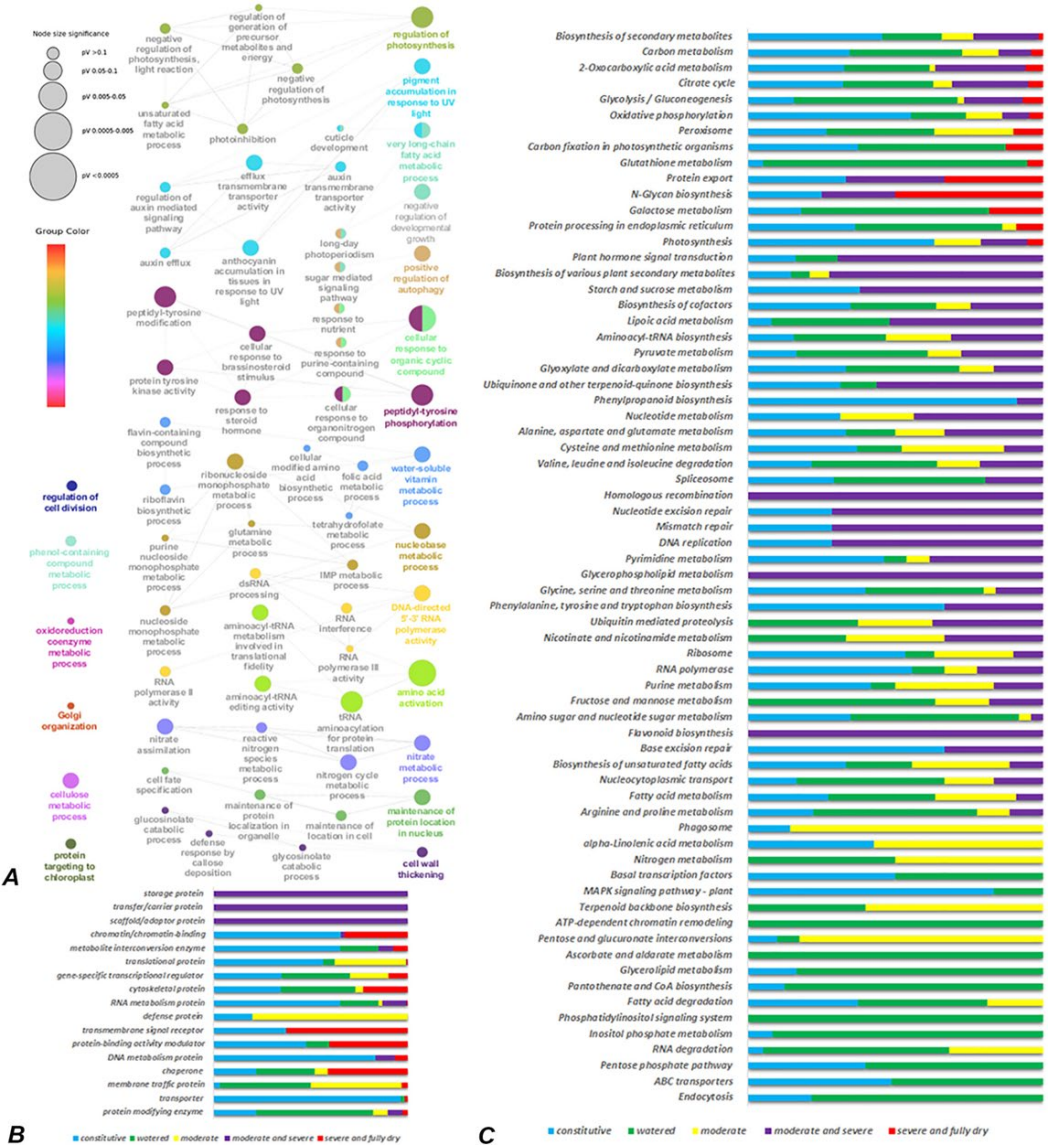
Functional annotation of genes corresponding to identified THSs. **A** Functionally organized network of the enriched biological processes. Node size represents significance and colour bar shows the connectivity of groups. **B** Protein classes found in various stages of stress, presented by percentage. C) KEGG pathway enriched in various stress stages, presented by percentage

To evaluate the cellular functions of genes associated with the identified THSs, we performed functional annotation for their protein classes and the biological processes and metabolic pathways in which they may participate (Fig. 4). Various processes were enriched and functionally connected to form networks, including negative regulation of photosynthesis and related processes such as regulation of generation of precursor metabolites and energy, photoinhibition, oxidoreduction coenzyme metabolic process; sugar mediated signalling pathway, auxin mediated signalling pathway, and related processes such as auxin efflux, auxin transmembrane transporter activity, cellulose metabolic process, cell wall thickening, very long-chain fatty acid metabolic process; regulation of cell division and negative regulation of developmental growth; cellular response to brassinosteroid stimulus and to organic cyclic compound; RNA polymerase III activity and related processes such as tRNA aminoacylation for protein translation; maintenance of protein location in nucleus, maintenance of protein localization in organelle, protein targeting to chloroplast, Golgi organization; positive regulation of autophagy, and response to nutrient (Fig. 4A).

Further, to evaluate the physiological and biochemical changes affected by the differentially identified THSs, we performed enrichment analysis on protein classes and pathways (Fig. 4B and 4C). According to distribution of THSs in Fig. 3B, we defined several stages of desiccation to access the dynamics of chromatin changes and the corresponding genes involved in different biological processes (Fig. 4A). These stages reflect the continuous changes in the chromatin accessibility during the transition from watered stage (C state) to moderate stage of desiccation (C and D1 states), from moderate to severe stage of desiccation (C, D1 and D2 states), and from severe to fully dry stage (D2 and D3 states), as well as constitutively accessible genes found in all stress states of plants (all states). Most of enriched protein classes and pathways represents THSs which were either constitutively accessed, or accessible in watered plants, including protein modifying enzymes and the pathways of endocytosis, ABC transporters, RNA degradation, inositol phosphate metabolism, pantothenate and CoA biosynthesis, ascorbate and aldarate metabolism and ATP-dependent chromatin remodelling (Fig. 4B and 4C). Specifically, at the beginning of desiccation (C + D1), the accessible sites detected by ATAC-seq were mostly associated to defense proteins, the translational proteins, membrane traffic protein, and gene-specific transcriptional regulators, and involved in the pathways of pentose and glucuronate interconversions, carbon metabolism, cysteine and methionine metabolism, biosynthesis of cofactors, oxidative phosphorylation, ribosome, peroxisome, glyoxylate and dicarboxylate metabolism, aminoacyl-trna biosynthesis, rna degradation, phagosome, biosynthesis of various plant secondary metabolites, citrate cycle, pyruvate metabolism, nucleotide metabolism, valine, leucine and isoleucine degradation, purine metabolism and alpha-linolenic acid metabolism. When stress became moderate and severe (D1 + D2) the accessible sites detected by ATAC-seq were related with DNA and RNA metabolism proteins and pathways of DNA replication and repair, plant hormone signal transduction and biosynthesis of secondary metabolites. Interestingly, storage proteins, carrier and adaptor proteins, scaffold/adaptor proteins, and pathways of Homologous recombination, Glycerophospholipid metabolism and Flavonoid biosynthesis were uniquely enriched in this stage. When stress became severe and plants were fully dried (D2 + D3) the accessible sites detected by ATAC-seq were mostly associated to transmembrane signal receptors, protein-binding activity modulators, chaperones, chromatin/chromatin-binding proteins, cytoskeletal proteins, and metabolite interconversion enzymes (Fig. 4B) and involved in N-glycan biosynthesis (a post-translational modification of glycoproteins), protein export, peroxisome, protein processing in endoplasmic reticulum, glutathione metabolism, and several primary metabolism pathways including carbon metabolism, oxidative phosphorylation, glycolysis/gluconeogenesis, citrate cycle, 2-oxocarboxylic acid metabolism as indicated by the KEGG analysis (Fig. 4C).

### Integration with RNA-seq data

**Fig. 5:**
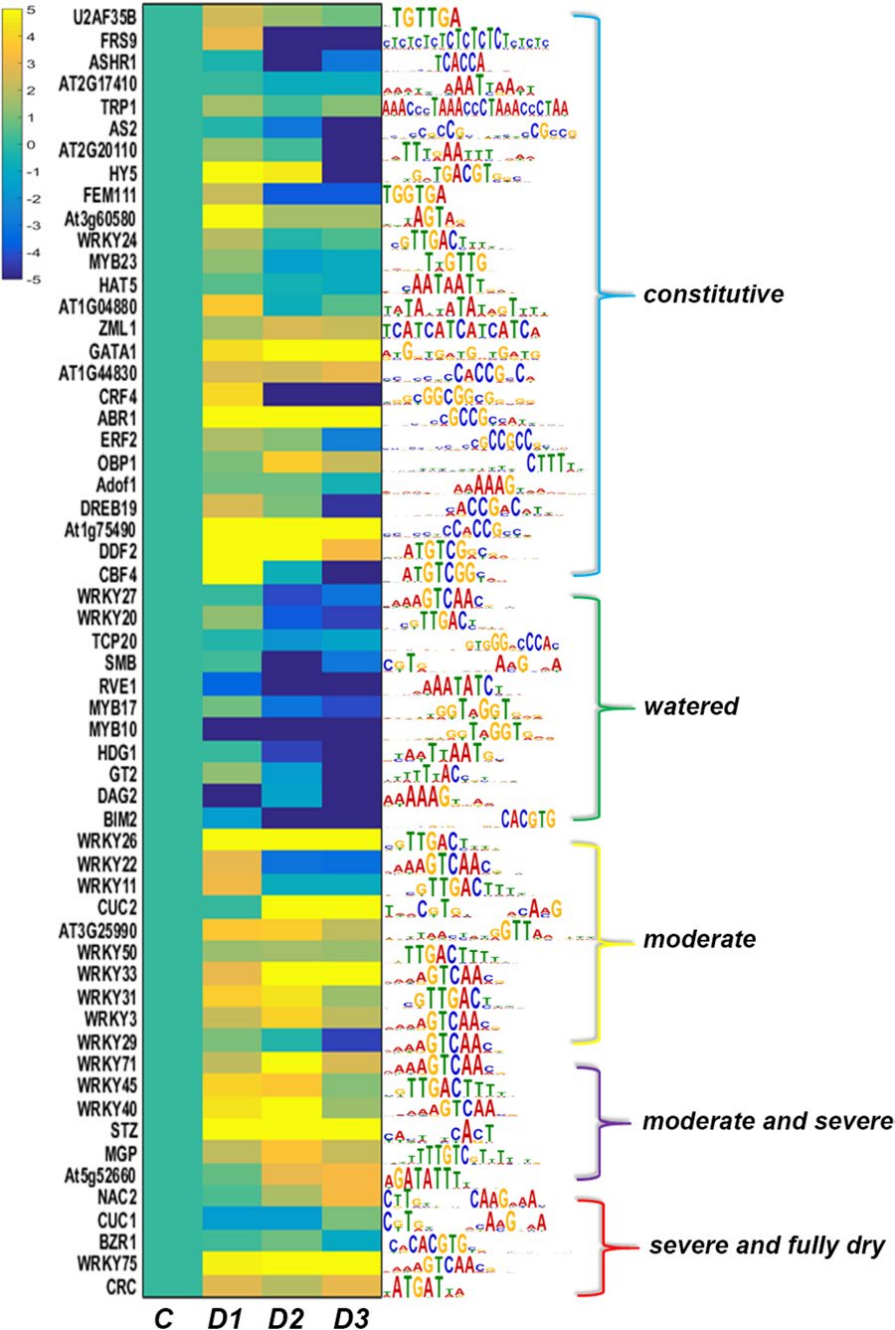
Expression of transcription factors related with the enriched motifs during desiccation stress in *H. rhodopensis*. Heatmap shows the fold change (FC) of peak counts from RNA-seq data under each stress stage compared to control state. The sequences of the motifs enriched from ATAC-seq data under different desiccation conditions are presented on right side of the heatmap

To evaluate the expression of genes corresponding to the identified THSs at different stress states, we integrate the results from ATAC-seq analysis with the previously published RNA-seq data from plants with same stress states [14]. First, we established a link of expression data of 58 transcription factors (TFs) from several families according to the motifs enriched from the promoter regions identified by ATAC-seq (Fig. 5; Additional file 7). These transcription factors were expressed at different levels during stress, and most of their expression patterns correlate positively or negatively with the accessibility of their binding motifs. In the group of TFs whose binding motifs were accessible in both watered and stressed conditions, i.e. in a constitutive manner, the expression of *HAT5* (*ATHB1*), *ADOF1*, and *At2g17410* (*ARID2*) were relatively stable, expression of *ASHR1* and *AS2* decreased at D1, D2 and D3, *GATA1*, *ZML1*, *DDF2*, *ABR1*, *At1g75490* (*DREBA2*), *AT1G44830* (*DREBA-5*), *ZML1*, and *OBP1* increased their levels at D1, D2 and D3. *HY5* and *DDF2* transiently increased their expression ratio in D1 and D2 state, and others including *DREB 19*, *CRF4*, *FRS9*, *ERF2*, *At3g60580* (*ZAT9*), *AT1G04880* (*HMGBD15*), *URAF358* (a putative gene in 3′ splice site RNA), *MYB23*, *CBF1*, *FEM111*, *AT2G20110* (*TCX6*) and *WRKY24* shows transiently increased levels in D1 state. Most of these TFs showed decreased expression in fully desiccated plants. Exclusively, all TFs whose binding motifs were accessible only in watered condition showed decreased expression in all states of desiccation stress, including a homeobox-leucine zipper factor (HDG1), two WRKYs (WRKY27 and WRKY20) and three MYBs (RVE1, MYB17 and MYB10). Interestingly, most of the TFs whose binding motifs were accessible in moderate dehydration state belongs to WRKY family. *WRKY26*, *WRKY50*, *WRKY33* and *WRKY3* showed increased levels at D1, D2 and D3 states, while *WRKY 22*, *WRKY 11* and *WRKY 29* shows transiently increased levels of expression only in D1 state and *WRKY31* shows transiently increased levels of expression only in D1 and D2 states. Besides, a NAC factor CUC2 was also found in this group, which showed increased levels at all stress states. Three WRKYs are among the 6 TFs whose binding motifs were found accessible in moderate and severe stress. Among them, *WRKY 45* and *WRKY 40* showed transiently increased expression at D1 and D2, while *WRKY71* increased at all stress states. Five TFs including a NAC2, CUC1, BZR1, WRKY 75 and CRC were found in the group of TFs whose binding motifs were accessible in severe and fully dry conditions of stress. Only *WRKY75*, *CRC* and *NAC2* showed increased expression in all drought states, while *CUC1* were transiently decreased at D1 and D2, and *BZR1* were transiently decreased at D3.

**Fig. 6:**
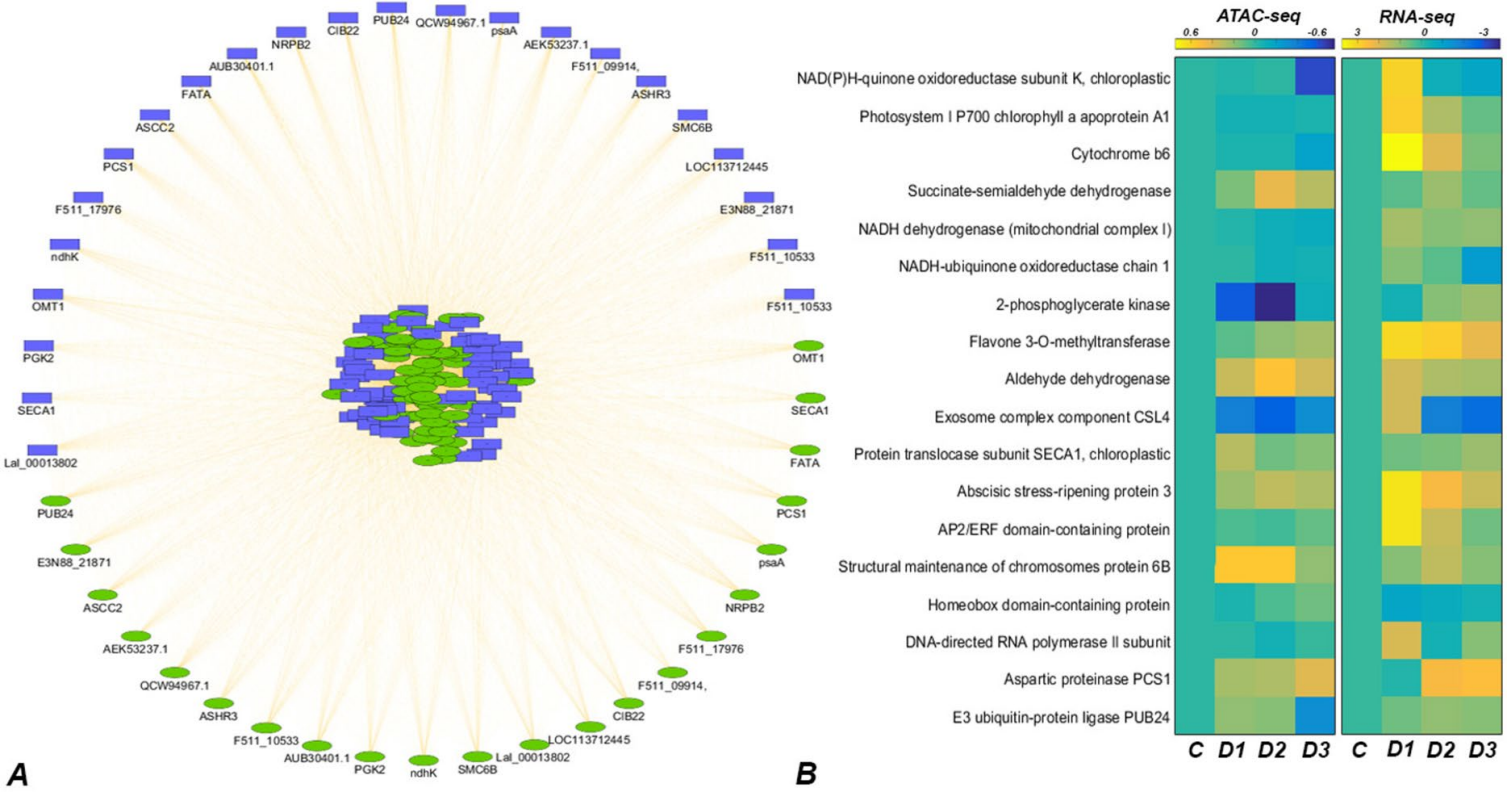
Correlation analysis between ATAC-seq and RNA-seq data. **A** Correlation network between peak counts of genes identified by ATAC-seq (blue rectangles) and RNA-seq data (green ovals). Each gene is represented twice, corresponding to peak counts from both analyses. Selected genes from different processes and pathways are presented. **B** Heat map of FC of peak counts of selected genes under each stress state compared to the control state for both analyses

Next, we evaluated the correlation between abundances of THSs and corresponding DEGs from RNA-seq data. As a result, a total of 526 genes from ATAC-seq, which were transposase accessible in both watered plants and desiccation-stressed plants, were mapped to the RNA-seq data and organize the count matrices as impute files in Cytoscape [36] for network correlation analysis. We were able to obtain a correlation network between 90 genes from both datasets (Fig. 6 A). Genes from photosynthesis, carbon metabolism, secondary metabolism, signalling, transport, chromosome maintenance and DNA metabolism and autophagy were represented (Fig. 6 B).

### THSs-linked differential gene expression associated with desiccation tolerance in *H. rhodopensis*

**Fig. 7:**
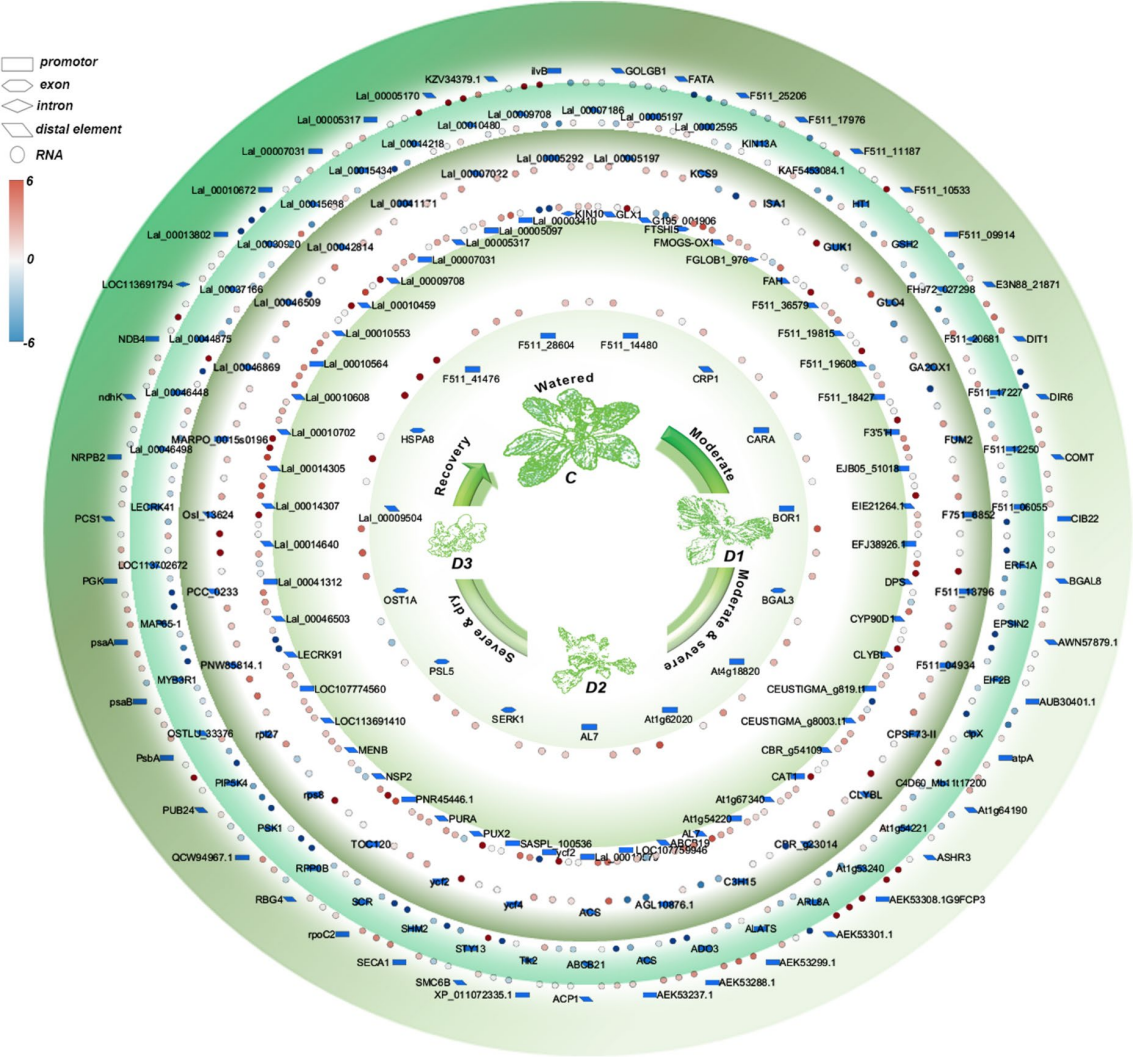
THSs and accumulation of transcripts of their corresponding genes. THSs are represented with different symbols in the top left. The log2 fold change (FC) of gene expression in D1, D2 and D3 states, compared to control (fresh state), is presented in a counter clockwise manner for each THS-corresponding gene. The outermost circle denotes the constitutively accessible genes, while the subsequent circles, as indicated in the innermost schematic diagram, represent particular stages of stress

We further analysed the THSs which were transposase accessible in certain stages of desiccation stress with their corresponding genes with expression information under desiccation [14]. As a result, a total of 192 genes accessible in various stress conditions exhibited differential expression ratios to control stage (Fig. 7, Additional file 8). THSs corresponding genes involved in Photosynthesis, Biosynthesis of secondary metabolites, Carbon metabolism, Glycolysis / Gluconeogenesis, Glyoxylate and dicarboxylate metabolism, Fatty acid degradation, Oxidative phosphorylation, DNA metabolism, Protein export and Starch and sucrose metabolism, such as *OMT1, ilvB, COMT, FUM2, BGAL1, FATA, ndhK, CIB22, NDB4, atpA, atpB, PUB24, psaA, psbC* and *psbD,* have been found in all stages of stress with different levels of transcription. Transcriptionally accessible genes only in watered plants include *ABCB21, ACS, ADO3, ALATS, ARL8A, clpX, EIF2B, EPSIN2, ERF1A, HT1, KIN13A, LECRK41, MAP65-1, OSTLU_33376, PIP5K4, PSK1, RPP0B, SCR, SHM2, STY13* and *Tlk2*. Genes with accessibility in moderate stress are involved in photosynthesis, cellular transport, cell wall organization, peroxisomes, TCA cycle and starch and sucrose metabolism and autophagy, including *Lal_00046869*, *Lal_00041171*, *Lal_00005292*, *ycf4*, *CPSF73-II*, *TOC120*, *F511_04934*, *rps8*, *ACS*, *GLO4*, *CLYBL*, *F511_13796*, *ISA1*, and *F751_6852*. During moderate and severe stages, transposase accessible genes include *Lal_00007031, CBR_g54109, Lal_00010564, FGLOB1_976, Lal_00010553, LOC113691410, Lal_00014305, CEUSTIGMA_g8003.t1, G195_001906, Lal_00005317, F511_19608, Lal_00010608, CAT1, F511_36579, CLYBL, EIE21264.1, ycf2 EFJ38926.1* and *EJB05_51018*, involving in Carbon metabolism, Biosynthesis of secondary metabolites, Glutathione metabolism, Peroxisome, Oxidative phosphorylation, Citrate cycle, 2-Oxocarboxylic acid metabolism, chromatin-binding, cell wall, DNA repair and autophagy. Genes annotated to *At4g18820*, *F511_28604*, *BGAL3*, *SERK1*, *PSL5*, *F511_41476*, *HSPA8* and *OST1A* involving in DNA repair, chromatin binding, transmembrane signal receptor, chaperons, N-Glycan biosynthesis and protein export were shown to be transposase accessible in severe and fully dry stage.

## Discussion

Climate change triggered reasonable and drastic increase of studies on the mechanisms behind the remarkable desiccation tolerance of the resurrection plants. In recent years, several resurrection species from Gesneriaceae family, native to the Balkan Peninsula and China, have been extensively studied. Although genome sequencing is currently available only for *D. hygrometricum* [9], various transcriptomics, proteomics and metabolomics studies have been performed to decipher the physiological and molecular mechanisms underlying their exceptional desiccation tolerance [12–15, 18, 37, 38]. Besides, it has been hypothesised that desiccation tolerance in resurrection plants may have evolved by activation of seed specific genetic elements in the vegetative tissues, which makes their genomic regulation different from that of desiccation sensitive species. Indeed, resurrection plants employ some common components of signal transduction pathways and epigenetic regulation via DNA methylation and histone modifications that are similar to seeds [39, 40]. The silencing of these genetic elements in non-resurrection plants is obviously related with imprinting events in development of plant embryos caused by transposon or repeat sequences leading to DNA methylation and silencing of neighbouring genes in vegetative tissues [41]. Epigenetic regulation is not only involved in seed imprinting, but also in priming of seeds, plant stress memory and cross-stress tolerance in non-resurrection and resurrection plants and is of particular interest for future research to improve desiccation sensitive species and especially crops [42]. In this respect, the epigenomic studies of resurrection and non-resurrection species could further link the to-date available physiological and omics studies not only in the context of genetic maps and syntenic blocks, but also with epigenetic regulation of stress memory and imprinting evolution related with desiccation tolerance of resurrection plants.

Here, using the genome of *D. hygrometricum* as a reference, we provide the first (genomic) evidence for the dynamic accessibility of chromatin regions including the regulatory elements during desiccation of *H. rhodopensis*. ATAC-seq analysis had been widely used to address the chromatin regulation of gene expression from other plant species [26–28]. The major technical challenge is to isolate intact nuclei from partially and fully desiccated plant samples. We developed a relatively fast and reproducible procedure for isolation of intact nuclei from *H. rhodopensis* tissues with different water contents (Fig. 1). It (The procedure) incorporates commercially available reagents and previously published protocols for other plant species [43]. We overcome the lacking genome sequencing information for *H. rhodopensis* utilizing the genome sequences of *D. hygrometricum*—phylogenetically close species with available genome sequencing data. Based on the relatively high gene homology between *H. rhodopensis* and *D. hygrometricum*, our analysis revealed that the proximal promotors and distal intergenic regions contained the highest number of identified THSs, followed by intragenic exon and intron regions (Figs. 2 and 3). According to the distribution of chromosome accessible sites in the classified stress states of *H. rhodopensis* (Fig. 3B), we defined several stages of the dynamic changes of chromatin accessible regions and the corresponding genes involved in different biological processes (Figs. 4 and 7). Unsurpressingly, we identified also constitutively accessible genes that are present in all stress stages. The previously developed and used for metabolomics and transcriptomic studies non-destructive method [13, 14] classifying stress states in *H. rhodopensis* allowed us to directly integrate the identified THSs and enriched motifs with the expression of their corresponding genes as well as the TFs that bind to them. Our results revealed a good correlation between ATAC-seq and RNA-seq data for constitutively accessible genes, as well as that between the accessibility of enriched motifs in different desiccation stages and the expression of their binding TFs (Figs. 5 and 6). Specifically, most genes accessible only in the watered stage exhibit a continuous decrease in expression during desiccation (Fig. 7, Additional file 8). A subset of these genes demonstrates a transient increase in expression in the D1 state, followed by decreasing when desiccation becomes severe and extreme. This could be attributed to the occurrence of posttranscriptional regulation of gene expression and the dynamic change in mRNA degradation rates. Furthermore, genes corresponding to THSs found in moderate stress show predominantly higher expression in D1 stage compared to that in the C, D2 and D3 (Fig. 7, Additional file 8). Moreover, genes corresponding to the THSs found in later stages of desiccation exhibited mostly increased expression in D1, D2 and D3 states compared to control state, with varying degrees of magnitude (Fig. 7, Additional file 8).

We also identified multiple types of TF-binding sites (*cis*-elements) within the promoter regions annotated by THSs identified under various stress stages (Figs. 3C and 5). These findings suggest a potential role of the TFs in regulating the desiccation-triggered gene expression alteration through their interaction with respective *cis*-elements in the open chromatin. Among the TFs whose binding elements were enriched based on the THSs identified as being constitutive accessible during desiccation are DREB1, DREB2, and ABR1. These crucial signalling components for plant adaptation to drought stress [44, 45], were with increased expression during desiccation. Two GATA family TFs, GATA1 and ZML1, were found to be overexpressed during desiccation. GATA family transcription factors are widely found in animals and plants. In animals they are predominantly related with repression of apoptosis and erythropoiesis, while in plants they are involved in regulation of broad range processes such as photomorphogenic growth, chlorophyll biosynthesis, chloroplast development, photosynthesis, and stomata formation [46]. Overexpression of GATA1 could be related with the transcriptional regulation of nitrogen assimilation as reported in Arabidopsis [47]. But more interestingly, ZML1 is involved in photoprotection of plants, exposed to light intensities that exceed the electron utilization capacity of the chloroplast [48, 49]. The up-regulation of these factors (Fig. 5) during desiccation are likely to associate with the activation of desiccation signal pathways and protective adaptations. Conversely, TFs including FRS9, FEM111/AGL80, ASHR1, AT2G20110/TCX6 and AS2 exhibited a decrease in expression under desiccation. FRS (FAR1-RELATED SEQUENCE) is a conserved TF family in plants known to regulate plant growth and development through transposable elements in *A. thaliana* [50]*.* FEM111/AGL80 is involved in central cell and endosperm development in Arabidopsis [51]. AT2G20110/TCX6 (Tesmin/TSO1-like CXC domain-containing protein) and AS2 are two transcriptional repressors that play roles in the repression of maintenance of DNA methylation and silencing of genes related with leaf polarity establishment and shoot apical meristem development [52]. ASHR1 was recently reported to regulate gene expression during anaerobic germination in rice [53]. The down-regulation of these factors (Fig. 5) during desiccation possibly contributes to the stress-induced growth retardation. In agreement, we successfully identified constitutive THS-corresponding genes that are involved in various cellular processes and pathways during desiccation (Fig. 4). Significant correlation between chromosomal accessibility and expression of matched corresponding genes in RNA-seq data was obtained for constitutively opened chromatin regions (Fig. 6). Genes from photosynthesis—Photosystem I P700 chlorophyll *a* apoprotein A1 and NAD(P)H-quinone oxidoreductase subunit K, chloroplastic related with maintenance of cyclic electron flow as photoprotective response in *H. rhodopensis* [13] increased their expression during desiccation (Figs. 6B and 7, Additional file 8). Genes related with signalling and chromatin folding like abscisic stress-ripening protein 3 and structural maintenance of chromosomes protein 6B showed also good correlation with THSs and increased gene expression during desiccation. Chloroplastic transporter protein translocase subunit SECA1, which is essential for functioning of photosynthetic machinery [54] was also with increased expression. We found that the accessibility of promotor for Succinate-semialdehyde dehydrogenase involved in TCA cycle and catabolism of 4-aminobutyrate (GABA) accumulated during drought stress in many plants [55] correlate with the overexpression of this enzyme during desiccation. E3 ubiquitin-protein ligase PUB24 involved in phagosome also increases accessibility of promotor sites in D1 and D2 states parallel with the increasing of associated DEGs. We found also increased expression of an ELIP gene with constitutively accessible promotor in agreement with previous studies with *H. rhodopensis* and other resurrection and non-resurrection plants [15, 56, 57].

We found that all TFs that are predicted to bind motifs accessible only in watered condition showed decreased expression in all states of desiccation, including three MYBs and two WRKYs (Fig. 5). MYB family factors, typically involved in plant growth and development, appeared to regulate negatively these processes during desiccation stress in *H. rhodopensis*. For example, decreasing of RVE1, a member of MYB family, is associated with the auxin and circadian signalling networks [58]. This finding supports the previously reported downregulation of auxin signalling receptors [14]. WRKY factors are one of the largest families of transcriptional regulators in plants. They modulate many processes in growth, development and senescence, and responses to abiotic and biotic stress by binding to W-box TTGAC(C/T) in the promoter of its target genes and activate or inhibit the expression of downstream genes [59–61]. In cotton, WRKY27 coordinates the senescence regulatory pathway [62]. The accessibility of w-box in watered plants and the decrease of WRKY27 and WRKY20 in desiccated plants supports the negative regulation of senescence in *H. rhodopensis* under desiccation, as suggested previously [15]. In addition to these TFs, according to RNA-seq data, expression of most genes corresponded to the watered stage-unique THSs decreased during desiccation (Fig. 7, Additional file 8). Such genes encode 65-kDa microtubule-associated protein, phosphatidylinositol 4-phosphate 5-kinase, LRR receptor-like serine/threonine-protein kinase, clathrin interactor EPSIN 2, phytosulfokines 1, transcription factor MYB3R-1, checkpoint protein, and ethylene-responsive transcription factors. These proteins are involved mainly in growth and development, and their downregulation is consistent with the reduced level of chromatin accessibility (Fig. 3A), the inhibition of plant growth, and the above-mentioned decrease of growth -regulating TFs under desiccation.

Unlike the diverse types of TFs that bind to constitutively accessible or watered plants-specifically accessible elements, almost all of the TFs whose binding motifs were accessible under moderate stress belong to WRKY family (Fig. 5). Moreover, the majority of these WRKY TFs are found to be increasingly expressed during all stress stages, highlighting their significant roles in regulating desiccation-induced gene expression. On the other hand, a wide array of genes implicated in various biological processes—photosynthesis, cellular transport, cell wall organization, peroxisomes, TCA cycle and starch and sucrose metabolism were identified in association with THSs during this stage of desiccation. Notably, their expression patterns exhibited distinct differences (Figs. 4 and 7, Additional file 8). Among these genes, *TOC120,* encoding translocase of chloroplast 120 – a GTPase involved in protein precursor import into chloroplasts, displayed elevated expression in all stages of desiccation. Photosystem I assembly protein Ycf4, which is pertinent to the stability and functioning of PSI [63], kept high levels of mRNA during stress. The high expressions of these genes facilitate the transport of chloroplastic proteins and protection of photosystem I during desiccation stress in *H. rhodopensis.* Moreover, two isoforms of fumarate hydratase, succinate dehydrogenase and peroxisomal (S)-2-hydroxyacid oxidase, key players in the glyoxylate cycle, peroxisomes and TCA cycle, were also with elevated expression during desiccation. This observation aligns with the previously reported function of TCA cycle in ensuring carbon and energy supply during the later stages of desiccation, alongside the well preservation of mitochondria structure and function in *H. rhodopensis* [13–15, 64]. In contrast, the Isoamylase gene exhibited a transient increased expression in the D1, followed by a decrease in the D2 and D3 stages. This expression pattern is consonant with the early starch consumption observed in *H. rhodopensis* soon after desiccation start [18]. Additionally, the increased expression of pectin esterase during desiccation is consistent with the previously reported accumulation of its protein in *H. rhodopensis* [15]. Lastly, flavonoid 3',5'-hydroxylase, which is involved in the synthesis of secondary metabolism, has also been found to exhibit increased levels of transcriptions [14]. These findings suggest a potential transcriptional regulation of rapidly induced genes in *H. rhodopensis* in response to stress.

Under moderate and severe desiccation, a reduction in accessible motifs was observed. These motifs are putative binding sequences of TFs including WRKY 40, WRKY45, WRKY71, RVE6 (At5g52660), STZ and MGP (Fig. 5). All of the matched DEGs corresponding to these TFs were increased during desiccation. Among these, TFs, WRKYs and MGP exhibited the highest mRNA levels in the D2 state, while STZ and RVE6/At5g52660 remained high in the D3 state. Previous studies have demonstrated that WRKY40, WRKY45 and WRKY71 can transactivate or interact with key components of ethylene, gibberellin and abscisic acid signalling pathways under abiotic stress conditions [59]. RVE6 is a major member of the REVEILLE clock genes, which inhibit plant growth [65]. STZ and MGP are members of a motif for Cys-2/His-2-type zinc-finger TFs. MGP plays a role in root patterning [66]and overexpressing STZ showed growth retardation and tolerance to drought stress [67]. Correspondingly, THSs, found in this stage, influenced various genes involved in Carbon metabolism, Biosynthesis of secondary metabolites, Glutathione metabolism, Peroxisome, Oxidative phosphorylation, Citrate cycle, 2-Oxocarboxylic acid metabolism, chromatin-binding, cell wall, DNA repair and autophagy (Figs. 4 and 7, Additional file 8). Like under moderate stress, THSs for genes such as succinate dehydrogenase, citrate synthase, oxoglutarate dehydrogenase and two malate synthases were found at moderate and severe stage of desiccation accompanied with the increasing of their corresponding DEGs as previously published [14], thus supporting the proposed role of TCA cycle in desiccation tolerance of *H. rhodopensis* [15]. THS-corresponding genes encoding pectinesterase (a key enzyme involved in cell wall organization), several transporters, such as protein translocase subunit SecA, cationic amino acid transporter, sodium transporter HKT1-like and protein Ycf2, were found to accumulate in this stage. A key regulator of phenylpropanoid pathway—4-coumarate-CoA ligase [68], also increased in this stress stage, indicating that the carbon pull is directed also toward synthesis of secondary metabolites. THSs for DNA-repair complementing XP-A cell gene and Scaffold protein Nfu/NifU were also found, supporting the previously published data for DNA repair and chromosome maintenance in *H. rhodopensis* [14, 15]. THSs for SNF1-related protein kinase involved in autophagy [69] were found as well supported by increased expression of corresponding DEG.

At late stage of desiccation, we identified lowest number of unique THSs. One possible reason is that many of them were not annotated in databases (data not shown). This reflects the need for future work on investigation of chromosomal regulation when the genome sequence data is available for *H. rhodopensis.* Nevertheless, we were able to enrich motifs corresponding to WRKYs and a NAC family TF which corresponds with higher expression of TFs during desiccation (Fig. 5). Concurrently, genes for transmembrane signal receptor, chaperons such as heat shock 71 kDa and proteins with functions in chromatin binding and posttranslational modification of glycoproteins by N-Glycan biosynthesis were identified by THSs (Figs. 4 and 7, Additional file 8). STICHEL-like proteins have been reported as key regulators of trichome branch number in Ararbidopsis [70] and could be potentially linked with the significantly increased formation of adaxially positioned trichomes during desiccation in *H. rhodopensis*, as well as in two other closely related resurrection species, e.g., *R. serbica* and *R. myconi* [71]. Beta-galactosidases, known to participate in the degradation of cell wall polysaccharides [72, 73], and thus could involve in cell wall rearrangements, as observed previously in other resurrection plants [74]. Beta-1,3-Glucanases, including glucan 1,3-alpha-glucosidases, are engaged in a broad range of processes like cell division, trafficking, biotic and abiotic stresses and seed maturation [75]. Often their expression supports accumulation of other proteins such as chitinases, peroxidases, thaumatin-like proteins, which also increased in *H. rhodopensis* during desiccation [15]. Notably, among the genes corresponding to the last stage-specific THSs, SERK1, a member of somatic embryogenesis receptor kinases (SERKs), exhibited elevated gene expression. SERKs serve as pivotal regulators in a broad range of processes including brassinosteroid signalling, male sporogenesis, vascular development and floral abscission as well as a negative regulators of leaf senescence in Arabidopsis [76]. SERK1 have been reported to be directly participate in stomatal patterning and exert negative regulation of stomatal development [77, 78]. Another key regulator of stomatal closure in response to ABA signalling in guard cells, OST1 (Open stomata 1/SnRK2.6/SnRK2E) [79], was found to show a marked increase in expressed [79]. Thus, our findings of THSs for these two genes during severe and full desiccation, coupled with their significant overexpression, strongly support the presumptions of conserved regulation in stomata closure during drought stress in resurrection plants and non-resurrection plants [80].

## Conclusion

In recent years, drought tolerance became a main target of modern plant science and the efforts for sustainable agriculture. Desiccation tolerant species, known as resurrection plants, have also been intensively studied, as suitable models of tolerance to extreme stress. Here, we performed, for the first time, ATAC-seq with resurrection plants, in particular – *H. rhodopensis*, to identify the genome regulatory sites of genes involved in various processes influenced, involved or resulting in desiccation tolerance, such as photosynthesis, carbon metabolism, synthesis of secondary metabolites, cell signalling and transcriptional regulation, cell growth, cell wall, stomata conditioning, chaperons, oxidative stress and autophagy. By utilizing our previously published transcriptomic and proteomics data, we integrated the identified THSs at various stages of desiccation with the expression and translation of corresponding genes and the putative binding transcription factors. Our approach provides a layout for further studies on plant stress tolerance—epigenetic control, comparative genomics and potential for application of New Genomic Technics (NGT) in crop improvement.

## Materials and methods

### Plant materials and desiccation stress

*Haberlea rhodopensis* plants with same size and development of rosettes from same biotope were harvested by Petko Vasilev Mladenov from Rhodope mountains near Bachkovo Monastery (N41°59.290’; E 24°52.260’). Voucher specimens (So 105,793) from same locality were deposited in the Herbarium of Sofia University St. Kliment Ohridski (SO). The plants were then adapted in pots with controlled irrigation and environment at 24 °C a 16:8 day and night photoperiod, 40% to 60% relative air humidity, and a photon flux density of 36 µmol m^−2^. s^−1^ for about a month. Desiccation stress was imposed by withholding water and dynamics of the stress was evaluated as described in Mladenov et al., 2015 [13]. Briefly, a self-organized map (SOM) was used to classify leaf samples according to photosynthetic performance evaluated by fast fluorescence measurements. Fluorescence measurements were performed using Handy-Plant efficiency analyser (handy-PEA) (Hansatech Instrument Ltd., King's Lynn, Norfolk, PE30 4NE, UK) device in dark and light adapted plants and recordings were organized and classified in SOM as described in details [13]. Samples in fully watered state and several states of desiccation, such as moderate-D1 (50% RWC), severe-D2 (20% RWC), and fully dry-D3 (6% RWC) according to predefined nodes of SOM were used for further analyses.

### Nuclei isolation

Nuclei isolation from fully watered plants (C), moderate desiccated (D1), severe desiccated (D2) and fully desiccated plants (D3) was performed with CelLytic™ PN Isolation/Extraction Kit (Sigma Aldrich) according to manufacturer’s instructions with some modifications. Briefly, 5 g leaves from different states of desiccation were ground in liquid nitrogen with mortar and pestle and transferred in tubes. Homogenization of powder with nuclei isolation buffer (NIB) was facilitated with ultra-sonication bath two times for 5 s and homogenate was filtered through the filter mesh and centrifuged 1,260 × *g* for 10 min at 4^◦^C. Pellet was resuspended and homogenized with Dounce homogenizer and then 10% TRITON™ X-100 was added to a final concentration of 0.3% (v/v). After 30 min incubation on ice, cell integrity was evaluated by microscope and homogenate was loaded on the top of a 3 ml of 60% Percoll solution overlaid onto 3 ml of 2.5 M sucrose and centrifuge with swinging bucket rotor at 1,260 × *g* for 30 min at 4^◦^C. Applying the standard procedure of CelLytic™ PN extraction kit to *H. rhodopensis* was not efficient enough to obtain sufficiently pure nuclei, especially from dried plants, according to microscopic observations (data not shown). Therefore, after lysing the cell membranes, we applied two additional centrifugation steps with different Percoll™ layers as recommended by Sikorskaite et al. 2013 [43]. The effective resuspension of cell pellets by Dounce homogenizer and cell lysis also significantly improved the purity of isolated nuclei after Percoll™ centrifugation steps. Percoll layer between sucrose layer and homogenate was collected according to Sikorskaite et al. 2013 [43] and transferred to new tube; nuclei were washed with NIB buffer and pelleted by centrifugation at 8,000 × *g* for 5 min. Pellet was resuspended in 2 ml NIB buffer and applied on another 3 ml of 35% Percoll cushion followed by centrifugation at 1,800 × *g* for 20 min. The resulting pellet was resuspended NIB buffer and washed by centrifugation at 8,000 × *g* for 5 min. Finally, nuclei were resuspended in 500 µl storage buffer and stored in aliquots at -80^◦^C. Nuclei isolation was performed with three biological replicates for each state of stress. To evaluate the yield from each sample, nuclei were counted after DAPI staining on fluorescent microscope using standard procedure with hemocytometer plate. Images from microscope were processed and nuclei were counted using MatLab (Mathworks, Natick, MA, USA) software with standard algorithm for object counting from images.

### Library construction for ATAC-seq analysis

ATAC-seq library construction was essentially performed according to Bajic et al. 2018 [26] using ATAC-seq kit (Active Motif). For each treatment, three biological replicates were used and 60,000 nuclei from each were pelleted by centrifugation. Tagmentation reaction and purification of tagmented DNA were performed according to manufacturer instructions of ATAC-seq kit. For amplification of library, barcoded indexed primers based on Illumina’s Nextera adapters were used. Barcodes for each combination of indexed primers are given for each sample in Additional file 1. PCR was performed using the following program on a thermal cycler (with a heated lid): 72 °C 5 min; 98 °C for 30 s; 10 cycles of: 98 °C for 10 s, 63 °C for 30 s, 72 °C for 1 min; Hold at 10 °C. After PCR, the exact number of cycles was evaluated by qPCR according to Bajic et al., 2018 [26]. Subsequently, ATAC-seq libraries were purified using SPRI Beads according to manufacturer instruction and their concentrations were measured using Qubit. The quality and length of amplified libraries were visualized after electrophoretic separation on 2% agarose gel (Additional file 9).

### Sequencing and analysis of ATAC-seq data

ATAC-seq libraries were submitted to BGI for sequencing. Sequencing using the HiSeqX10 platform was conducted in the 150 base pairs paired-end (PE) mode, followed by read trimming to 100 bp PE for further analysis. ATAC-seq data processing and alignment were processed using the Harvard pipeline with some modifications (https://informatics.fas.harvard.edu/ atac-seq-guidelines.html). Raw sequencing data were firstly preprocessed using trim_galore (https://www.bioinformatics.babraham.ac.uk/projects/trim_galore/) to remove the Illumina Nextera Transposase adapter as well as sequences with low base quality sequence with “-q 25 –phred33 –length 35 -e 0.1” parameter. After trimming process, FastQC v0.12.1 (https://www.bioinformatics.babraham.ac.uk/projects/fastqc/) was used to assess general sequence quality and evaluate proper adapter trimming. All ATAC-seq reads were then aligned to the genomes of the *D. hygrometricum* [9] using Bowtie2 (version 2.3.5) with “-p 36 –very-sensitive” options [81]. Sambamba [82] were used to mark and remove duplicates using the markdup tool with “–overflow-list-size 600,000” options. Samtools [83] was used to sort and obtain uniquely mapped reads using “-f 2 -q 30” options. Aligned, deduplicated BAM files were transformed into a binary format known as bigWig using bedtools [84] for visualization purpose. Peak calling was executed for each sample using the MACS2 version 2.2.6 callpeak command with the following parameters “-g mm –nomodel –shift -100 –extsize 200” [85]. Peaks for all tissues were then merged together into a standard peak list. Only peaks reproducible in at least two technical replicates (minimal overlap 50%) were further considered for downstream analyses.

### Promoter motif enrichment analysis

The promoter sequences (3,000 bp upstream of the start codon) of the genes associated with all annotated peaks were collected from the genome of *D. hygrometricum* [9], a close relative resurrection species in the same tribe in Gesneriaceae with available genome sequencing information, and submitted to the Discriminative Regular Expression Motif Elicitation (DREME) [86]. The most overrepresented motifs in these genes were identified using default parameters. The identified DNA Motifs were then subjected to a homology search using the motif comparison tool SEA [86] to identify the best matching motifs.

### Clustering, functional annotation of ATAC-seq peaks and integration with RNA-seq data

To identify differences of highly accessible chromatin regions between desiccation treatments, we used annotation files and the fold change count peak matrix with all replicates derived from previous section, which was summarized by hierarchical cluster analysis (HCA) and visualized with heatmap with MatLab (Mathworks, Natick, MA, USA) software according to standard algorithms. Annotated peaks were further subjected to functional enrichment using GO and KEGG databases [87–89]. GO functional enrichment and visualization of terms in functionally grouped network was performed by the ClueGO plugin [90] for Cytoscape software [36]. To map and visualize all identified genes and ontology relationships, a general mapping method was used and Bonferroni correction was omitted. Subsequently, the clustered and annotated differentially accessible chromatin regions were enriched in GO by protein classes and KEGG databases using the available online tools in databases with their corresponding gene names and KO identificators. To integrate ATAC-seq data with the previously published RNA-seq data performed at the same stress stages of *H. rhodopensis* [14], queries from both analyses were unified by their gene names and UniProt entries and fold change count peak matrix was combined with RNA-seq FPKM results. Correlation of abundances of reads between genes found in both analyses in each state of stress was evaluated and visualized by correlation network construction with clusterMaker plugin for Cytoscape software [36]. Correlation network was set up by the Elucidean squared distance of means of reads abundances between treatments for both analyses. Connected nodes represented correlation between genes with means closer to 0,01. Only connections between ATAC-seq and RNA-seq were taken in account.

### Supplementary Information

**Additional file 1. **Samples and PCR primers. Sample numbers correspond to each stress stage with the corresponding replicates (3). Indexed Primers are based on Illumina’s Nextera adapters as follow: Index primer 1 (i7)-CAAGCAGAAGACGGCATACGAGAT[i7barcode] GTCTCGTGGGCTCGG and Index primer 2 (i5) AATGATACGGCGACCACCGAGATCTACAC [i5barcode] TCGTCGGCAGCGTC. Barcodes for each combination of indexed primers are given for each sample.

**Additional file 2. **Plot of fluorescence versus cycle from qPCR Library Amplification check of bulked DNA library pool from all samples.  

**Additional file 3. **Sequence quality Raw reads produced from sequencer contain adapters, unknown or low quality bases.

**Additional file 4. **Quality and distribution reads. Left picture, base percentage distribution along reads the sample; right picture, distribution of qualities along reads of the sample.

**Additional file 5. **TSS plots and heat maps for each numbered replicate corresponding to Additional file 1, showing numbers of mapped sequences from the corresponding genes from genome of *D. hygrometricum*. 

**Additional file 6. **Identified THSs and corresponding genes from *H. rhodopensis* at different desiccation stages.

**Additional file 7. **Motif enrichment statistics.

**Additional file 8. **Gene annotation of selected THSs with the representative sequence reads and log2 FC of their corresponding DEGs.

**Additional file 9.** Raw image of visualization of ATAC-seq libraries.

## Data Availability

Data supporting the results reported in the article were deposited in the NCBI with BioProject identifier PRJNA1021767. (https://www.ncbi.nlm.nih.gov/bioproject/?term=PRJNA1021767).

## Abbreviations

ATAC-seq: Assay for Transposase Accessible Chromatin with high-throughput sequencing
THS: Transposase hypersensitive site
TSS: Transcription start site
