# Supplementary material for: Dynamics of chromatin accessibility and genome wide control of desiccation tolerance in the resurrection plant Haberlea rhodopensis
Source: BMC Plant Biol. 2023 Dec 19;23:654. doi: 10.1186/s12870-023-04673-2 (PMC10729425; doi:10.1186/s12870-023-04673-2)
Supplement: Supplementary file 1 — Additional file 1. Samples and PCR primers. Sample numbers correspond to each stress stage with the corresponding replicates (3). Indexed Primers are based on Illumina’s Nextera adapters as follow: Index primer 1 (i7)-CAAGCAGAAGACGGCATACGAGAT[i7barcode] GTCTCGTGGGCTCGG and Index primer 2 (i5) AATGATACGGCGACCACCGAGATCTACAC [i5barcode] TCGTCGGCAGCGTC. Barcodes for each combination of indexed primers are given for each sample. [file 12870_2023_4673_MOESM1_ESM.docx]

| sample N | state | I7 index | I5 index | [I7] barcode | [I5] barcode |
| --- | --- | --- | --- | --- | --- |
| 1 | C-1 | 2 | 2 | CTAGTACG | CTCTCTAT |
| 2 | C-2 | 2 | 3 | CTAGTACG | TATCCTCT |
| 3 | C-3 | 2 | 1 | CTAGTACG | TAGATCGC |
| 4 | D1-1 | 1 | 1 | TCGCCTTA | TAGATCGC |
| 5 | D1-2 | 1 | 2 | TCGCCTTA | CTCTCTAT |
| 6 | D1-3 | 1 | 3 | TCGCCTTA | TATCCTCT |
| 7 | D2-1 | 4 | 4 | GCTCAGGA | AGAGTAGA |
| 8 | D2-2 | 4 | 3 | GCTCAGGA | TATCCTCT |
| 9 | D2-3 | 4 | 2 | GCTCAGGA | CTCTCTAT |
| 10 | D3-1 | 3 | 3 | TTCTGCCT | TATCCTCT |
| 11 | D3-2 | 3 | 1 | TTCTGCCT | TAGATCGC |
| 12 | D3-3 | 3 | 2 | TTCTGCCT | CTCTCTAT |

**Additional file 1.** Samples and PCR primers. Sample numbers correspond to each stress stage with the corresponding replicates (3). Indexed Primers are based on Illumina’s Nextera adapters as follow: Index primer 1 (i7)-CAAGCAGAAGACGGCATACGAGAT[i7barcode] GTCTCGTGGGCTCGG and Index primer 2 (i5) AATGATACGGCGACCACCGAGATCTACAC [i5barcode] TCGTCGGCAGCGTC. Barcodes for each combination of indexed primers are given for each sample.
