# Supplementary material for: Dynamics of chromatin accessibility and genome wide control of desiccation tolerance in the resurrection plant Haberlea rhodopensis
Source: BMC Plant Biol. 2023 Dec 19;23:654. doi: 10.1186/s12870-023-04673-2 (PMC10729425; doi:10.1186/s12870-023-04673-2)
Supplement: Supplementary file 2 — Additional file 2. Plot of fluorescence versus cycle from qPCR Library Amplification check of bulked DNA library pool from all samples. [file 12870_2023_4673_MOESM2_ESM.docx]

**
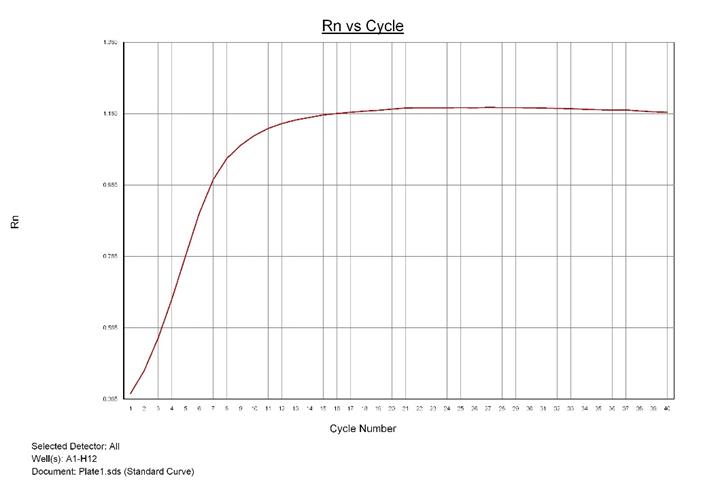
**

**Additional file 2.** Plot of fluorescence versus cycle from qPCR Library Amplification check of bulked DNA library pool from all samples**.** No additional cycles were needed with the used PCR protocol.
