# Supplementary material for: Dynamics of chromatin accessibility and genome wide control of desiccation tolerance in the resurrection plant Haberlea rhodopensis
Source: BMC Plant Biol. 2023 Dec 19;23:654. doi: 10.1186/s12870-023-04673-2 (PMC10729425; doi:10.1186/s12870-023-04673-2)
Supplement: Supplementary file 3 — Additional file 3. Sequence quality Raw reads produced from sequencer contain adapters, unknown or low quality bases. [file 12870_2023_4673_MOESM3_ESM.docx]

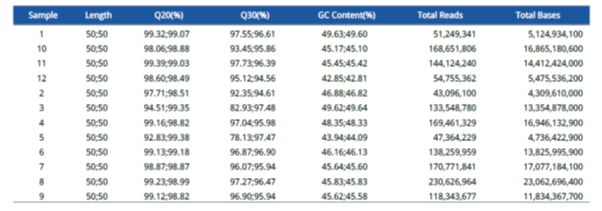


**Additional file 3**. Sequence quality Raw reads produced from sequencer contain adapters, unknown or low quality bases.
