# Supplementary material for: Dynamics of chromatin accessibility and genome wide control of desiccation tolerance in the resurrection plant Haberlea rhodopensis
Source: BMC Plant Biol. 2023 Dec 19;23:654. doi: 10.1186/s12870-023-04673-2 (PMC10729425; doi:10.1186/s12870-023-04673-2)
Supplement: Supplementary file 4 — Additional file 4. Quality and distribution reads. Left picture, base percentage distribution along reads the sample; right picture, distribution of qualities along reads of the sample. [file 12870_2023_4673_MOESM4_ESM.docx]

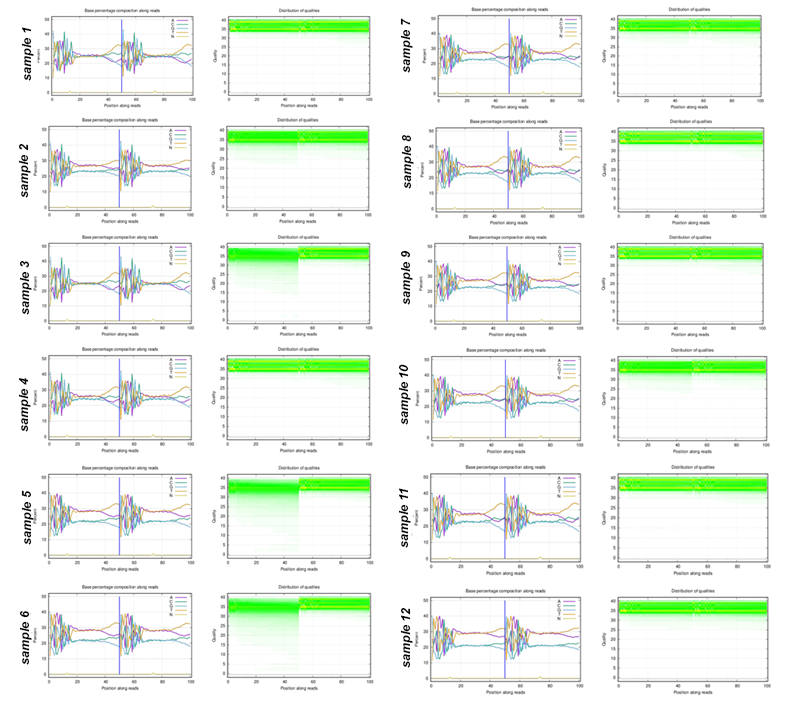


**Additional file 4.** Quality and distribution reads. The distribution of base percentage and qualities along reads in data filtering are shown (If a sample has multiple lanes, only one of them will be displayed). Left picture, base percentage distribution along reads the sample; right picture, distribution of qualities along reads of the sample.
