# Supplementary material for: Dynamics of chromatin accessibility and genome wide control of desiccation tolerance in the resurrection plant Haberlea rhodopensis
Source: BMC Plant Biol. 2023 Dec 19;23:654. doi: 10.1186/s12870-023-04673-2 (PMC10729425; doi:10.1186/s12870-023-04673-2)
Supplement: Supplementary file 5 — Additional file 5. TSS plots and heat maps for each numbered replicate corresponding to Additional file 1, showing numbers of mapped sequences from the corresponding genes from genome of D. hygrometricum. [file 12870_2023_4673_MOESM5_ESM.docx]

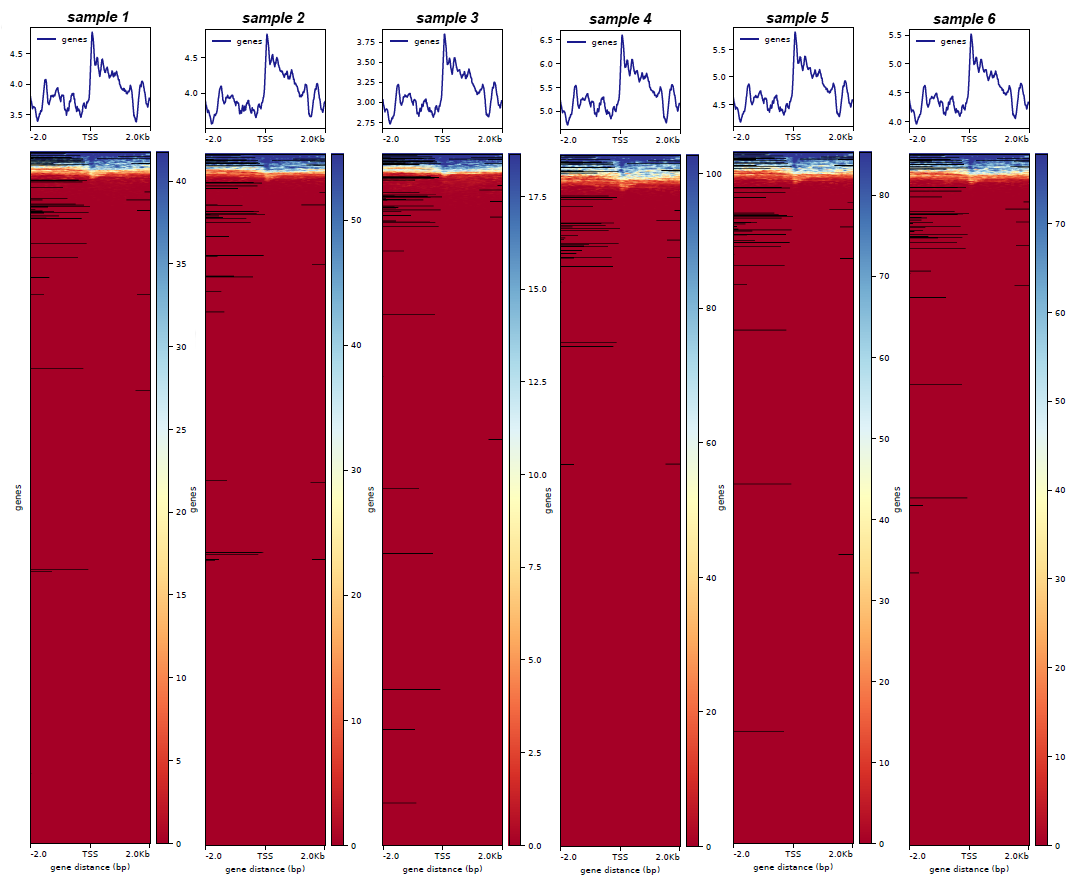


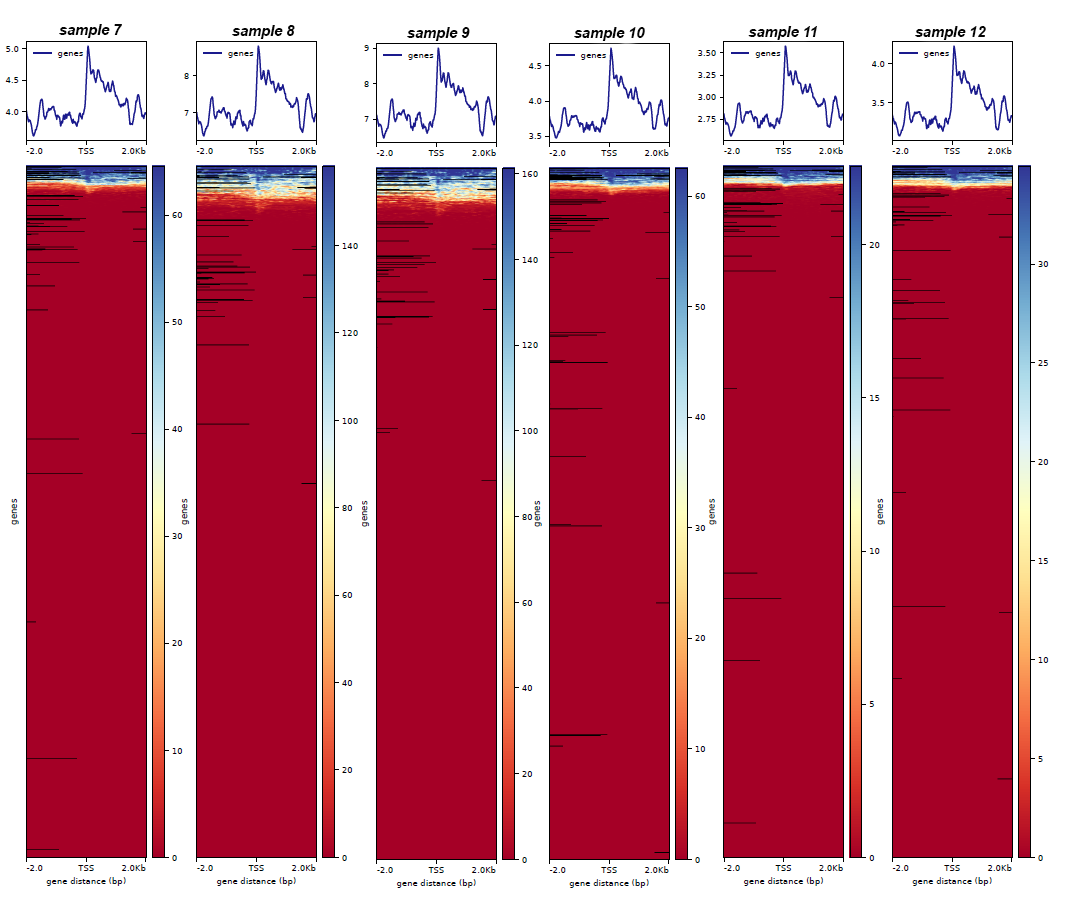


**Additional file 5.** TSS plots and heat maps for each numbered replicate corresponding to Additional file 1, showing numbers of mapped sequences from the corresponding genes from genome of *D. hygrometricum*.
