# Supplementary material for: Dynamics of chromatin accessibility and genome wide control of desiccation tolerance in the resurrection plant Haberlea rhodopensis
Source: BMC Plant Biol. 2023 Dec 19;23:654. doi: 10.1186/s12870-023-04673-2 (PMC10729425; doi:10.1186/s12870-023-04673-2)
Supplement: Supplementary file 9 — Additional file 9. Raw image of visualization of ATAC-seq libraries. [file 12870_2023_4673_MOESM9_ESM.docx]

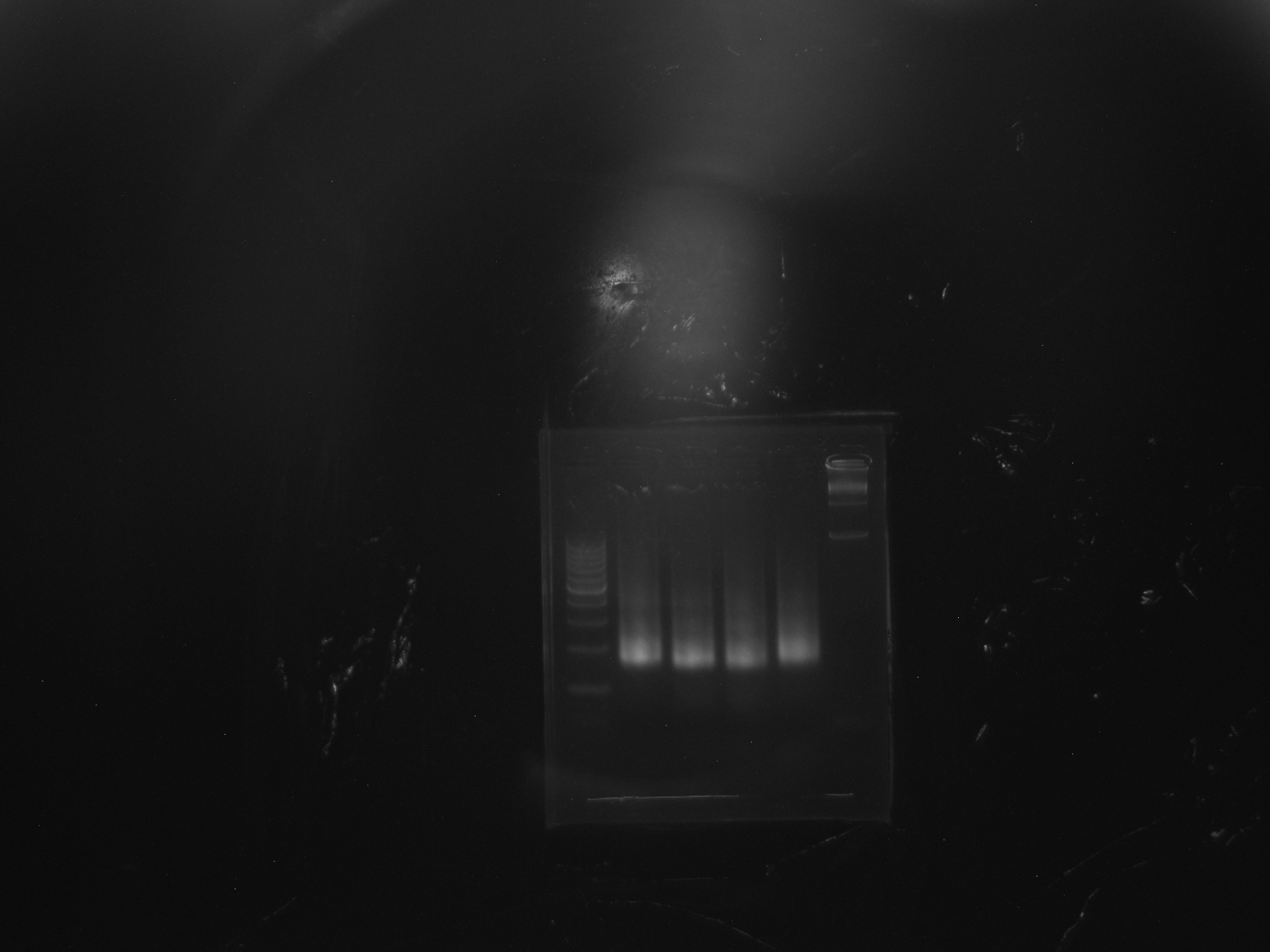


**Additional file 9.** Raw image of visualization of ATAC-seq libraries. Starts from left to right: M (100 bp); C; D1; D2; D3. Last start is 1000bp ladder and don’t represent in figure of manuscript
